# Kisspeptin and Endometriosis—Is There a Link?

**DOI:** 10.3390/jcm13247683

**Published:** 2024-12-17

**Authors:** Blazej Meczekalski, Agata Nowicka, Stefania Bochynska, Aleksandra Szczesnowicz, Gregory Bala, Anna Szeliga

**Affiliations:** 1 Department of Gynecological Endocrinology, Poznan University of Medical Sciences, 60-535 Poznan, Poland; 2 UCD School of Medicine, University College Dublin, D04 V1W8 Dublin, Ireland

**Keywords:** kisspeptin, endometriosis, hypothalamic-pituitary-gonadal axis, gonadotropin-releasing hormone (GnRH), KISS1R receptor, reproductive health, hormonal regulation, matrix metalloproteinases (MMPs)

## Abstract

This article presents a narrative review that explores the potential link between kisspeptin—a key regulator of the hypothalamic-pituitary-gonadal axis—and the pathogenesis of endometriosis. Kisspeptin plays a significant role in regulating reproductive functions by modulating the release of gonadotropin-releasing hormone (GnRH), which in turn stimulates the secretion of luteinizing hormone (LH) and follicle-stimulating hormone (FSH). Recent studies suggest that kisspeptin may also impact peripheral reproductive tissues and influence inflammatory processes involved in the development of endometriosis. Altered kisspeptin signaling has been associated with the abnormal hormonal environment observed in endometriosis, which affects menstrual cycles and ovarian function. Research indicates that women with endometriosis exhibit altered levels of kisspeptin and its receptor, KISS1R, in both eutopic and ectopic endometrial tissues, suggesting a role in disease progression, particularly in tissue invasion and lesion formation. Kisspeptin’s role in regulating matrix metalloproteinases (MMPs), enzymes essential for tissue remodeling, further supports its potential contribution to the pathophysiology of endometriosis. Moreover, kisspeptin-based therapeutic strategies are currently under investigation, with the aim of providing targeted treatments that reduce the side effects commonly associated with existing therapies. Despite promising findings, further research is needed to fully understand the mechanisms by which kisspeptin influences endometriosis.

## 1. Introduction—The Hypothalamic-Pituitary-Ovarian Axis as a Main Regulator of Reproductive Functions and the Regulatory Role of GnRH

The process of human reproduction is governed by complex regulatory mechanisms, the foremost of which is the hypothalamic-pituitary-gonadal (HPG) axis.

The hypothalamus produces releasing hormones, among which gonadotropin-releasing hormone (GnRH) is a critical regulator of reproductive hormonal function. Synthesized in approximately 1000–1500 hypothalamic neurons located in the medial preoptic area (POA) and the arcuate/infundibular nucleus, GnRH secretion originates in the median eminence. It is then released into the hypophyseal portal circulation, where it exerts its effect on the anterior pituitary by binding to its G protein receptor on the surface of gonadotrope cells [1,2].

GnRH receptor regulation is achieved through mechanisms such as ligand-induced internalization of receptors, changes in receptor number, or alterations in the activity of signaling pathways downstream of GnRHR [3]. The expression of GnRHR is modulated via a feedback loop by ovarian steroid hormones, with estradiol shown to reduce GnRHR number in a dose- and time-dependent manner [4].

Two modes of GnRH secretion have been identified, pulsatile and surge [5]. The surge mode occurs in the preovulatory phase, characterized by a consistent and stable concentration of GnRH in the portal circulation [6]. Conversely, the pulsatile fluctuation of GnRH secretion varies physiologically over the female reproductive cycle, differentially promoting the synthesis and release of LH and FSH [7]. FSH is preferentially stimulated at low GnRH pulse frequencies, whereas LH is preferentially stimulated at high frequencies [2,8]. Kisspeptin has emerged as a key stimulatory regulator of GnRH secretion, a topic that will be explored in the following section.

Endometriosis is a complex disease, which is caused by abnormal growth of endometrial tissue outside the uterus. It affects about 5–10% of women of reproductive age. Endometriosis is associated with symptoms worsening the health and well-being of patients. Those are severe pelvic pain, dysfunction of the organs of the pelvic cavity, infertility, and secondary mental issues [9,10,11].

The accurate path of progression of endometriotic lesions is still unknown, and several mechanisms are suspected of being the main cause of developing ectopic implants, including Mullerian residuals, lymphatic or vascular invasion, and coelomic metaplasia. 

The most widely accepted is Sampson’s retrograde menstruation theory, which suggests the endometrial cell backflow in fallopian tubes and implantation to the peritoneal cavity. Although endometriosis is a benign disorder, it also has characteristics similar to malignities, like adhesion, invasion, and localization on ectopic tissues [12].

Kisspeptins are hormones of the RF-amid family that interact with matrix metalloproteinases (suspected of playing one of the key roles in the pathogenesis of endometriosis) in endometrial tissue to form focal adhesions to limit the tissue, limit trophoblast invasion during placentation, and suppress metastasis in various cancers. 

Kisspeptins are also known as neuropeptides, and they regulate the hypothalamic-hypophysial-gonadal axis are effective during puberty, and have major reproductive roles in the HPG axis. There are several studies suggesting that endometriosis may change kisspeptin metabolism. In this review, we summarize current knowledge on the association of endometriosis and kisspeptin and the HPG axis and potential usage of this in endometriosis treatment [10,13].

### Methods

This narrative review involved a comprehensive search of several major databases, including PubMed, ScienceDirect, Excerpta Medica Database, UpToDate, and the Cochrane Library. The exhaustive search strategy used a combination of MeSH terms alone or in combination, including the following: “endometriosis”, “kisspeptin”, “neurokinin b”, “KNDy”, and “hypothalamus”. Searches were conducted between 1 June 2024 and 29 June 2024. All identified publications in English up to July 2024 were thoroughly and critically appraised by the authors, with a particular and deliberate focus on studies directly related to the primary topic. Two authors independently reviewed the titles and abstracts, meticulously classifying them based on the strict inclusion criteria. Duplicates, conference proceedings, and editorial letters were excluded. Full texts of clinical studies, review articles, and meta-analyses were then systematically examined and evaluated. Additionally, reference lists of included articles were manually and carefully screened to identify further relevant studies.

## 2. Kisspeptin and KNDy Neurons—An Overview 

Kisspeptin (KP) has been identified as a crucial regulator of GnRH neurons, playing a pivotal role in orchestrating the onset of puberty and the maintenance of reproductive function. The inactivation of the *KISS1* or *KISS1R* genes, which encode for kisspeptin and its receptor, respectively, leads to delayed or absent puberty onset. Conversely, activating mutations of *KISS1R* can cause precocious puberty [14].

Discovered in 1996 by Lee et al. [15], kisspeptin is a neuropeptide that binds to a G-protein-coupled receptor known as KISS1R, a member of the rhodopsin family of G-protein-coupled receptors. Activation of the receptor stimulates phospholipase C, triggering secondary intracellular messengers, inositol trisphosphate and diacylglycerol, which control the intracellular secretion of calcium and the activation of protein kinase C [16].

Located primarily in the arcuate nucleus and, to a lesser extent, in the preoptic area [17], kisspeptin neurons also exist in the limbic and paralimbic regions, as well as in the placenta, pancreas, ovary, and liver [18]. The neurons in the arcuate nucleus co-express the neuropeptides neurokinin B (NKB) and dynorphin (Dyn), hence the designation KNDy neurons. Neurokinin B enhances synchronized KNDy neuronal activity through stimulatory Gq-coupled NK3 receptors to release kisspeptin, while Dynorphin A, secreted from KNDy neurons, terminates this activity via inhibitory Gi-coupled κ-opioid receptors [19].

KNDy neurons are instrumental in initiating GnRH and LH pulses across different species, with their communication being essential for the rhythmic interaction between GnRH and LH [20]. Furthermore, hypothalamic kisspeptin neurons are regulated by metabolic agents such as insulin, leptin, and ghrelin, linking metabolic health with energy balance and reproductive function [21].

**Figure 1:**
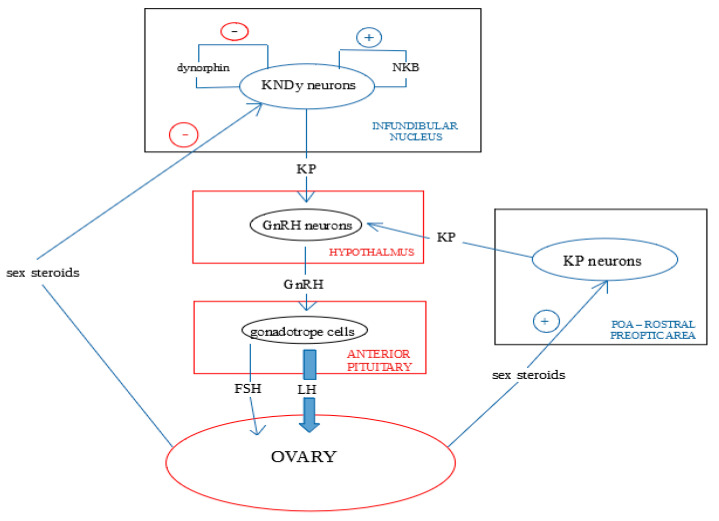
Kisspeptins impact on steroidogenesis through its influence on the hipothalmic-pituitary-ovarian axis. Based on [24].

Kisspeptin directly stimulates GnRH neurons, primarily through the activation of canonical transient receptor potential (TRPC) 4 channels and the inhibition of inwardly rectifying potassium channels. It also enhances ionotropic glutamatergic and GABAergic transmission to GnRH neurons, which depolarizes these neurons [22]. Moreover, recent studies have suggested that kisspeptin may be involved in additional pathways that integrate environmental cues with reproductive signals, further broadening its physiological relevance. Kisspeptin neurons project fibers into the POA, stimulating GnRH secretion (Figure 1), which in turn prompts the release of LH and FSH from the pituitary [18]. Inhibition of arcuate kisspeptin neurons has been shown to disrupt LH pulses and, consequently, the menstrual cycle and reproductive function [23]. These disruptions can lead to significant reproductive pathologies, emphasizing the importance of precise regulatory mechanisms and maintaining hormonal balance for overall health.

The activity of kisspeptin is modulated by sex steroids such as estrogen and progesterone, which regulate its activity through sex steroid receptors in both the anteroventral periventricular nucleus and the arcuate nucleus [25], as these neurons are sensitive to estradiol and express estrogen and express estrogen receptors [26]. Estradiol has a stimulatory effect on POA kisspeptin neurons but inhibits activity-regulated cytoskeleton-associated protein (ARC) kisspeptin neurons. During the LH surge, KISS1 expression increases in the POA but decreases in the ARC, indicating that negative feedback is controlled by ARC kisspeptin neurons, while positive feedback is regulated by POA kisspeptin neurons [18].

## 3. Endometriosis—Overview of Disease Pathomechanism

Endometriosis is a complex and debilitating gynecological condition affecting approximately one in ten women of reproductive age. It is characterized by the abnormal presence of endometrial tissue outside the uterus, often leading to significant impairment in quality of life and presenting considerable challenges in diagnosis and management. The disease manifests with symptoms like chronic pelvic pain, infertility, heavy menstrual bleeding, and gastrointestinal disturbances, all of which significantly impact patients’ daily activities and overall well-being, making even routine tasks difficult [9,10,11]. 

The definitive diagnosis and staging of endometriosis requires surgical visualization, typically performed via laparoscopy [27]. Histopathological assessment is essential for confirming the diagnosis and determining the disease stage. Various histopathological changes can be observed in tissue samples from patients with endometriosis, including hyperplasia, atypia, and, in some cases, malignant transformation. These changes are driven by local inflammatory processes, as well as vascular and hormonal factors, contributing to disease progression and cellular atypia. Additionally, these alterations may lead to malignant transformation, exacerbating symptoms such as pain and abnormal vaginal bleeding. Increased lesion proliferation is associated with altered tumor suppressor proteins and inhibition of cellular apoptosis [28].

The pathomechanism of endometriosis involves multiple physiological and molecular pathways that drive its development and progression. A deeper understanding of these mechanisms is essential for advancing treatment options and improving outcomes for patients with endometriosis.

The exact cause of endometriosis remains elusive; however, several theories have been proposed. The most widely accepted is Sampson’s theory of retrograde menstruation, which suggests that during menstruation, some shedding endometrial cells flow back through the fallopian tubes into the pelvic cavity, where they implant and grow [29]. Despite the occurrence of retrograde menstruation in many women, not all develop endometriosis, suggesting the involvement of additional factors such as genetic predisposition, immune system dysfunction, and environmental influences, potentially altering the body’s ability to recognize and destroy ectopic endometrial tissue [30].

The pathophysiology of endometriosis is multifaceted, involving immune system abnormalities, inflammation, angiogenesis, and estrogen dependency. Ectopic endometrial cells in endometriosis can evade the immune system, which would typically clear such misplaced tissue [31]. This evasion may result from altered immune surveillance or an immune response that promotes inflammation rather than resolution. Inflammation plays a crucial role in the pathomechanism of endometriosis, with the immune response to ectopic endometrial cells involving the release of pro-inflammatory cytokines, growth factors, and chemokines, promoting further immune cell recruitment, sustaining inflammation, and contributing to lesion and scar tissue formation [32]. Another significant aspect of the pathophysiology of endometriosis is angiogenesis, the formation of new blood vessels that facilitate lesion growth and proliferation by providing essential nutrients and oxygen [33]. The process is mediated by factors like vascular endothelial growth factor (VEGF), found in elevated levels in the peritoneal fluid of affected women, further promoting the spread of lesions [34]. Endometriosis’s strong estrogen dependency, with treatments often aiming to reduce estrogen production, highlights the estrogen sensitivity of ectopic endometrial tissue, similar to the uterine lining. Research indicates varying estrogen receptor expressions between normal and endometriotic tissues, with abnormal epigenetic changes such as DNA methylation contributing to disease development [35].

Recent studies have delved deeper into the molecular aspects of endometriosis, revealing alterations in gene expression and the involvement of various signaling pathways [35,36]. For example, the aberrant expression of genes related to cell adhesion, invasion, and survival facilitates the attachment and invasion of ectopic cells to the peritoneal lining. Moreover, dysregulation in pathways such as the Wnt/β-catenin pathway and the PI3K/AKT/mTOR pathway has been observed, which are crucial for cell proliferation and survival [37,38]. Emerging research also highlights the role of oxidative stress and mitochondrial dysfunction in endometriosis, contributing to cellular damage and promoting disease persistence. Understanding these cellular processes can guide the development of novel therapeutic approaches.

Endometriosis is characterized by a complex interplay of genetic, immunological, inflammatory, angiogenic, and hormonal factors. Its pathomechanism involves not only the implantation and growth of ectopic endometrial tissue but also significant alterations in the local and systemic environment, promoting its survival and expansion. Continued research into the molecular underpinnings of these processes is essential to develop more effective and targeted therapies, urgently needed to alleviate the burden of this condition. Advances in understanding the pathomechanism of endometriosis will likely pave the way for innovative treatments that can greatly improve the lives of millions of affected women worldwide.

## 4. Connections Between Kisspeptin Secretion and the Pathomechanism of Endometriosis

Recent research has increasingly focused on the role of hormonal regulation in the pathogenesis of endometriosis, with particular emphasis on kisspeptin, a key regulator of the hypothalamic-pituitary-gonadal (HPG) axis, which is responsible for regulating reproductive function [39]. The connection between the hypothalamic secretion of kisspeptin and other neurohormones and the pathomechanism of endometriosis is of particular interest to scientific society, as alterations in kisspeptin signaling may contribute to the development and progression of the disease. This is particularly important, as endometriosis contributes to profound worsening of quality of life; therefore, an explanation of its pathomechanism is critically important.

In the context of endometriosis, lower testosterone and higher estrogen levels have been associated with aberrant HPG axis function, where kisspeptin plays a significant role, resulting in lower body mass index (BMI), waist-to-hip ratio (WHR), and muscle mass [40,41]. The dysregulation of kisspeptin signaling could contribute to the disrupted reproductive hormonal environment seen in endometriosis. Such hormonal imbalances can affect the frequency and amplitude of GnRH and luteinizing hormone (LH) pulses, which are crucial for normal menstrual cycles and ovarian functions [42]. Lower LH and altered FSH levels seen in endometriosis mimic patterns seen in conditions such as premature ovarian insufficiency, thereby affecting follicular growth and overall reproductive health [43,44]. Such hormonal imbalances can severely impact the fertility and reproductive prospects of patients with endometriosis. 

**Figure 2:**
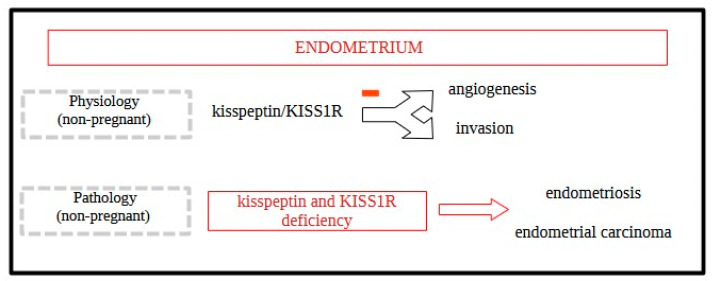
The roles of kisspeptin and KISS1R in physiological and pathological states of the endometrium of a non-pregnant women. Based on [49].

Furthermore, kisspeptin’s role extends beyond central hormonal regulation, which happens in the hypothalamus; it also influences peripheral reproductive tissues, including follicle maturation, endometrial receptivity, and potentially inflammatory pathways that contribute to the pathogenesis of endometriosis [45,46]. The altered expression of kisspeptin in the eutopic and ectopic endometrium of women with endometriosis highlights its potential role in disease progression, influencing tissue invasion and lesion establishment. Research indicates that women with endometriosis exhibit altered levels of kisspeptin and its receptor, KISS1R, particularly in eutopic and ectopic endometrial tissues, compared to healthy controls [47]. These abnormalities in kisspeptin signaling may disrupt the regulatory feedback loop necessary for the rhythmic secretion of GnRH, thereby impacting ovarian function and menstrual regularity. Both uterine- and placental-based kisspeptin systems inhibit trophoblast invasion and angiogenesis by downregulating matrix metalloproteinases (MMPs) and vascular endothelial growth factor A (VEGFA) [24]. Due to this, reduced kisspeptin levels may lead to the deregulation of matrix metalloproteinases, enzymes that facilitate tissue remodeling and invasion—critical processes in the establishment and progression of endometriotic lesions [48] (Figure 2).

Dysregulated kisspeptin signaling appears to be associated with reduced angiogenesis, decreased cytotrophoblast invasion, and increased trophoblast apoptosis, as lower levels of circulating kisspeptin have been observed in women with PE compared to normotensive pregnant controls. 

Thus, altered hypothalamic kisspeptin secretion not only disrupts endocrine balance but also contributes to the invasive characteristics of endometrial cells, enhancing the pathological landscape of a wide range of diseases, including endometriosis [24].

Understanding the link between kisspeptin and endometriosis underscores the importance of neuroendocrine regulation in reproductive pathologies. Further research into kisspeptin’s influence on the HPG axis, MMP activity, and inflammatory processes could pave the way for novel treatments, thereby improving the lives of millions of women worldwide. This neuroendocrine perspective may lead to innovative therapeutic approaches, potentially targeting this signaling pathway. Identifying how kisspeptin influences both hormonal and peripheral mechanisms may offer new insights into treatment strategies. These findings could inspire the development of pharmacological agents aimed at normalizing kisspeptin signaling, ultimately enhancing outcomes and quality of life for patients with endometriosis by targeting both systemic and local tissue effects of this pathway, with promising implications for future therapies.

## 5. Kisspeptin in Patients with Endometriosis—A Review of Studies

The association between kisspeptin (KP) serum levels and the occurrence of endometriosis (EM) is the subject of multiple research studies. It is still unclear if it can become a marker in detecting EM. It is being researched as a significant factor in the pathogenesis of EM as well as a potential treatment option. The relationship between kisspeptin (KP) serum levels and the occurrence of endometriosis (EM) has been the focus of numerous research studies. It remains unclear, however, whether kisspeptin can be utilized as a biomarker for detecting endometriosis. Kisspeptin is being investigated not only as a significant factor in the pathogenesis of endometriosis but also as a potential therapeutic target. At present, there are no pathognomonic laboratory findings for endometriosis.

Can kisspeptin become a viable marker for EM? 

In a recent systematic review, researchers comprehensively analyzed the available literature on endometriosis and its potential biomarkers [50]. Despite numerous studies and suggested markers, only four biomarkers were identified as having strong potential for diagnosing endometriosis: tumor necrotic factor α (TNF-a), matrix metalloproteinase-9 (MMP-9), tissue inhibitor of matrix metalloproteinase 1 (TIMP-1), and microRNA-451 (miR-451). Notably, kisspeptin was not included in this particular review of biomarkers.

Data on kisspeptin as a biomarker for endometriosis remains limited, and most studies are conducted on small patient cohorts. A study at the Elena Doamna Clinical Hospital of Obstetrics and Gynecology in Iasi, Romania, reported that mean serum kisspeptin levels in patients with endometriosis were 45% higher than those in a control group. No significant differences were observed in other hormonal measurements [51]. Although the study data suggest a very prominent difference in the level of serum kisspeptin levels, the study involved only eight women in each group, indicating a need for further research with larger sample sizes to validate these findings.

Another observational study by researchers at Ondokuz Mayis University Faculty of Medicine involved 40 women with endometriomas and a control group of women with no potential endometriosis symptoms. The study categorized the patients with endometriosis into groups with superficial endometriosis (SE) and deep infiltrating endometriosis (DIE) and found that both SE and DIE groups had significantly higher kisspeptin levels than the control group, although there was no significant difference between SE and DIE levels [10]. Despite the larger sample size, this study could not establish a significant determinative link between kisspeptin and endometriosis severity on account of the lack of differentiation between levels of kisspeptin in the SE and DIE groups.

A separate study aimed to examine the immunoreactivity of kisspeptin and its receptor, KISS1R, in both eutopic and ectopic endometrial tissues of women with and without endometriosis across the proliferative and secretory phases of the menstrual cycle. Tissue samples from 35 women with endometriosis and 14 control subjects were tested with KISS1 and KISS1R antibodies. Results indicated lower levels of KISS1 and KISS1R in the eutopic endometrium of patients with endometriosis compared to controls, suggesting that a deficiency in KISS1 and KISS1R may promote tissue invasion and contribute to endometriosis pathogenesis [47]. 

As a metastasis-suppressing protein, kisspeptin may act as a limiting factor in disease progression. The active forms of matrix metalloproteinase MMP-2 and MMP-9 are particularly important during the early stages of extragenital endometriosis development. KISS1, as a regulator of MMPs, can suppress protein transcription. It is believed that the suppression of MMPs is less effective in patients with endometriosis, leading to increased cell invasion. Elevated MMP-2 expression in the ectopic endometrium may indicate the aggressive progression of endometriosis, highlighting the need for targeted therapies [52].

These findings suggest that reduced expression of KISS1/KISS1R contributes to the pathogenesis of endometriosis by enhancing tissue invasion. This conclusion, however, is based on a sample of 40 women aged 23–38, and further research, on larger and more diverse groups, is needed to determine if similar patterns exist across other age groups.

Kisspeptin expressed in the endometrium binds to the KISS1R and triggers downstream molecular signaling pathways. These pathways have physiological functions in the endometrium, such as inhibiting invasion and angiogenesis. Kisspeptin may also inhibit the expression of stromal cell-derived factor-1 (SDF-1), which activates multiple signaling pathways associated with cell adhesion and migration, including FAK, PIK3, MAPK/ERK, and JAK/STAT, thereby suppressing cell invasion and migration [49].

Kisspeptin is proven to promote the development and function of endometrial glands in the uterus. It is also proven that turning off in adult mice decreases profoundly endometrial gland formation, and kisspeptin knockout mice’s uterus is almost completely devoid of endometrial glands [53]. Additionally, it is proven that endometrial adenogenesis and function are likely predominantly regulated by peripheral autocrine/paracrine KP signaling rather than ovarian steroid signaling [24].

The expression of KISS1/KISS1R in endometrial stromal cells is dependent on the phases of the menstrual cycle; expression of KP peptides is observed there only in the late secretory phase, while epithelial cells exhibit strong and consistent immunostaining for KP peptides and their receptor [54].

There is compelling evidence suggesting that kisspeptin is involved in metabolic pathways leading to elevated leptin levels in peritoneal and follicular fluids [52]. However, it remains unclear whether the peripheral kisspeptin system exerts only a local autocrine/paracrine effect or whether it also has direct feedback or modulatory “endocrine” effects on the central hypothalamic clock. Further investigation is needed to clarify this aspect.

## 6. Potential Therapeutic Target for Endometriosis—Dysregulated Kisspeptin Secretion

The unclear pathophysiology of endometriosis complicates predictions regarding the efficacy of emerging therapeutic options. Currently, there are two primary therapeutic approaches: hormonal treatments to alleviate symptoms and surgical removal of lesions. While surgery offers a potentially long-term solution, lesion recurrence rates are significant, ranging from 15% to 25% [55].

Existing pharmacological treatment consists of combined oral contraception, progestin-only pills, or GnRH analog with or without hormonal replacement therapy. As aromatase is the key enzyme for ovarian estrogen biosynthesis and there is evidence that endometriotic lesions express aromatase and are able to synthesize their own estrogens, aromatase inhibitors are also potent drugs that suppress estrogen synthesis via suppression of aromatase. They suppress the growth of endometriosis lesions and reduce the associated inflammation. This, in turn, significantly reduces their pelvic pain. Other medications are anti-inflammatory and immunomodulatory drugs and antioxidant drugs. Those medications has been investigated in the context of inflammation and have documented potential to restore redox balance in the follicular fluid microenvironment. Amongst emerging treatments, the most commonly mentioned are prostaglandin receptor inhibitors and stem cells [56,57]. Some of those medications, such as GnRH analogs or combined oral contraception, work by suppressing ovarian activity by turning off hypothalamic action or acting directly on steroid receptors and enzymes found in the endometrium and endometriotic lesions. Although these treatments can mitigate severe symptoms and improve quality of life, they often cause side effects, including reduced libido, increased body temperature, and loss of bone density [27]. Patients may experience hot flashes, night sweats, and other symptoms similar to the onset of perimenopause due to reduced estrogen levels [58]. 

The clinical effectiveness of managing endometriosis through GnRH suppression, combined with add-back therapy or selective progesterone receptor modulators [59] suggests that strategies based on partial gonadotropin suppression could have significant clinical relevance. However, selective progesterone receptor modulators (SPRMs) and pure progesterone receptor antagonists (PAs) can lead to severe estrogen deficiency, further contributing to vaginal atrophy, osteoporosis, and cardiovascular side effects.

In the context of endometriosis treatment, targeting partial suppression of the hypothalamus-pituitary-gonadal (HPG) axis through kisspeptin or neurokinin B antagonists could maintain therapeutic efficacy while avoiding drawbacks associated with GnRH. Approaches that do not rely on the complete suppression of the GnRH axis have clear clinical significance. Targeted, incomplete suppression of gonadotropins via kisspeptin may overcome the limitations of current GnRH analogs, although recent evidence suggests that KISS1 analogs might achieve complete hormonal suppression [60]. It is particularly important, as recently a whole new branch of medications, namely neurokinin B antagonists, started to be used in the treatment of menopausal symptoms. This is a whole new opportunity for patients suffering from vasomotor symptoms associated with menopausal transition with contraindications for hormonal replacement therapy. Those medications are tested and approved for treatment in the US and most European countries, which makes them a perfect target for examination of new indications for use.

The involvement of kisspeptin in nociceptive processes indicates a potential role for this neuropeptide in the treatment of endometriosis [11]. Endometriosis is characterized by both somatic and visceral pain mechanisms, manifesting in multiple common symptoms. This link could potentially be utilized in the pain management of endometriosis, although pain mechanisms in the disease are not yet fully understood, and causal relationships remain elusive. 

Given that the relationship between kisspeptin and endometriosis is a relatively new area of research, the use of kisspeptin analogs is currently hypothetical. Kisspeptin suppression is presumed to play a role in the pathogenesis and modification of the progression of endometriosis. However, several challenges exist with kisspeptin-based treatments, particularly concerning the need for therapeutic agents to cross the blood–brain barrier (BBB) to target central reproductive and non-reproductive pathways. Recent data suggests that different formulations of kisspeptin agonists and antagonists exhibit varying capabilities to cross the BBB when administered peripherally [61]. Such a treatment option is still only theoretical and needs to be further explored. 

## 7. Critical Analysis

In this narrative review, we presented the current advancements in understanding the relationship between endometriosis and KNDy neurons. However, information on this topic remains limited in the literature, underscoring the need for further research. We acknowledge that most existing studies in this area are non-randomized and involve only small patient cohorts. Consequently, the conclusions drawn from these studies are weak, and, as previously mentioned, randomized controlled trials are needed. This is particularly crucial given the potential therapeutic applications of kisspeptin and neurokinin B analogs for patients with endometriosis.

## 8. Conclusions

While kisspeptin holds promise as a potential biomarker for endometriosis, and further investigation into kisspeptin analogs is warranted, current knowledge and clinical application of potential biomarkers remain limited. Although endometriosis is a topic of significant clinical interest with numerous potential biomarkers across various studies, none are supported with sufficient evidence to be incorporated into official diagnostic guidelines at this time. We remain hopeful that future research will provide more robust data, allowing for a clearer picture of the potential of kisspeptin as a viable diagnostic tool or therapeutic option, thus contributing to the improvement of outcomes for patients affected by this condition.
